# Supplementary material for: Mechanism of GTPase activation of a prokaryotic small Ras-like GTPase MglA by an asymmetrically interacting MglB dimer
Source: J Biol Chem. 2024 Mar 18;300(4):107197. doi: 10.1016/j.jbc.2024.107197 (PMC11016934; doi:10.1016/j.jbc.2024.107197)
Supplement: Supporting Figures S1–S3 and Table S1 [file mmc1.pdf]

Supplementary Information

Mechanism of GTPase activation of a prokaryotic small Ras-like GTPase MglA by an asymmetrically interacting MglB dimer

Authors: Sukanya Chakraborty, Manil Kanade, and Pananghat Gayathri*

* Corresponding author: Pananghat Gayathri *

Indian Institute of Science Education and Research Pune, Dr Homi Bhabha Road, Pashan, Pune, India 411008.

Phone: +91-20-25908128

Email: gayathri@iiserpune.ac.in

Running title: GTPase activation by an asymmetrically interacting dimer

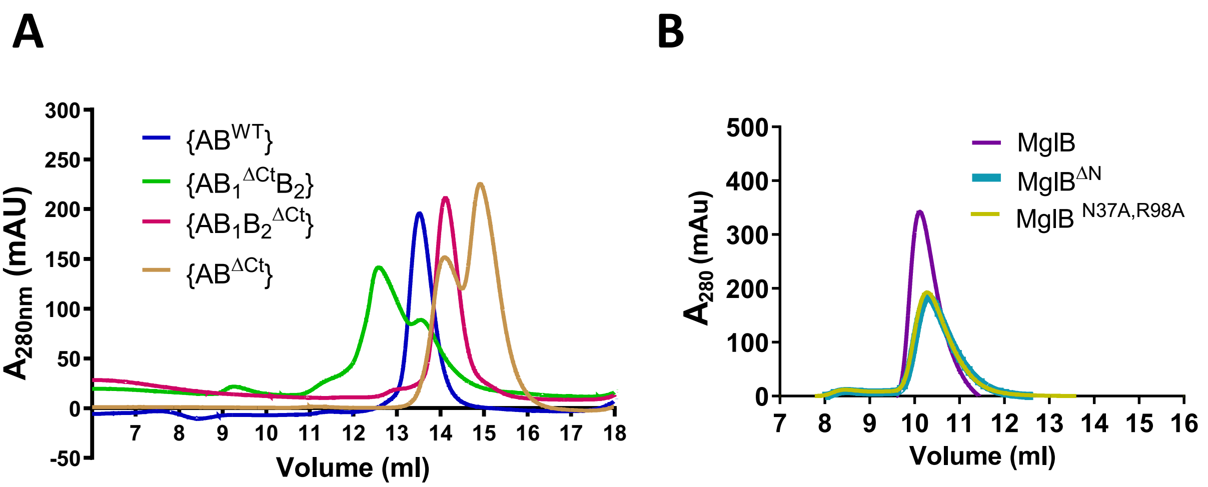

Supplementary Figure S1. Size exclusion chromatography for the linked and unlinked mutant constructs used in this study.

A: Analytical size exclusion chromatography (Superdex 200) profiles for the linked complexes: $\{AB^{WT}\}$ (blue), $\{AB_1B_2^{\Delta Ct}\}$ (magenta), $\{AB_1^{\Delta Ct}B_2\}$ (green) and $\{AB^{\Delta Ct}\}$ (orange).

B: Size exclusion chromatography (Superdex-75) profiles of mutants of MglB (purple), $MglB^{\Delta N}$ (cyan) and $MglB^{N37A,R98A}$ (ochre).

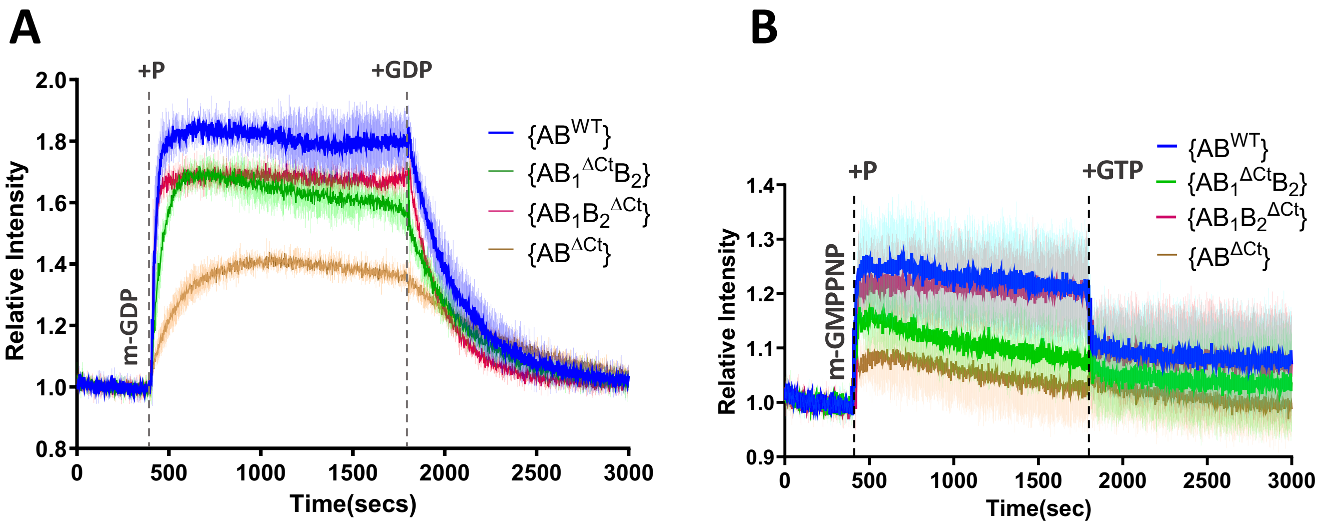

Supplementary Figure S2. Mean and standard deviation of the nucleotide exchange kinetics shown by the asymmetric linked complexes

A: Kinetics of increase in *mant*-GDP fluorescence (region labeled as *m*-GDP) upon adding linked complexes, {AB^{WT}} (blue), {$AB_1^{\Delta Ct}B_2$} (green), {$AB_1B_2^{\Delta Ct}$} (magenta), {$AB^{\Delta Ct}$} (brown) at 400 seconds (marked by the dashed line labeled '+P'), followed by a competition of *mant*-GDP by adding excess unlabeled GDP at 1800 seconds (marked by the dashed line labeled '+GDP'). The traces represent the mean values across replicates with the respective standard deviations shown in shaded lines.

B: Kinetics of increase in *m*-GMPPNP fluorescence (region labeled as *m*-GNP) upon adding linked complexes, {AB^{WT}} (blue), {$AB_1^{\Delta Ct}B_2$} (green), {$AB_1B_2^{\Delta Ct}$} (magenta), {$AB^{\Delta Ct}$} (brown) at 400 seconds (marked by the dashed line labeled '+P'), followed by a competition of *mant*-GMPPNP by adding excess unlabeled GTP at 1800 seconds (marked by the dashed line labeled '+GTP'). The traces represent the mean values across replicates with the respective standard deviations shown in shaded lines.

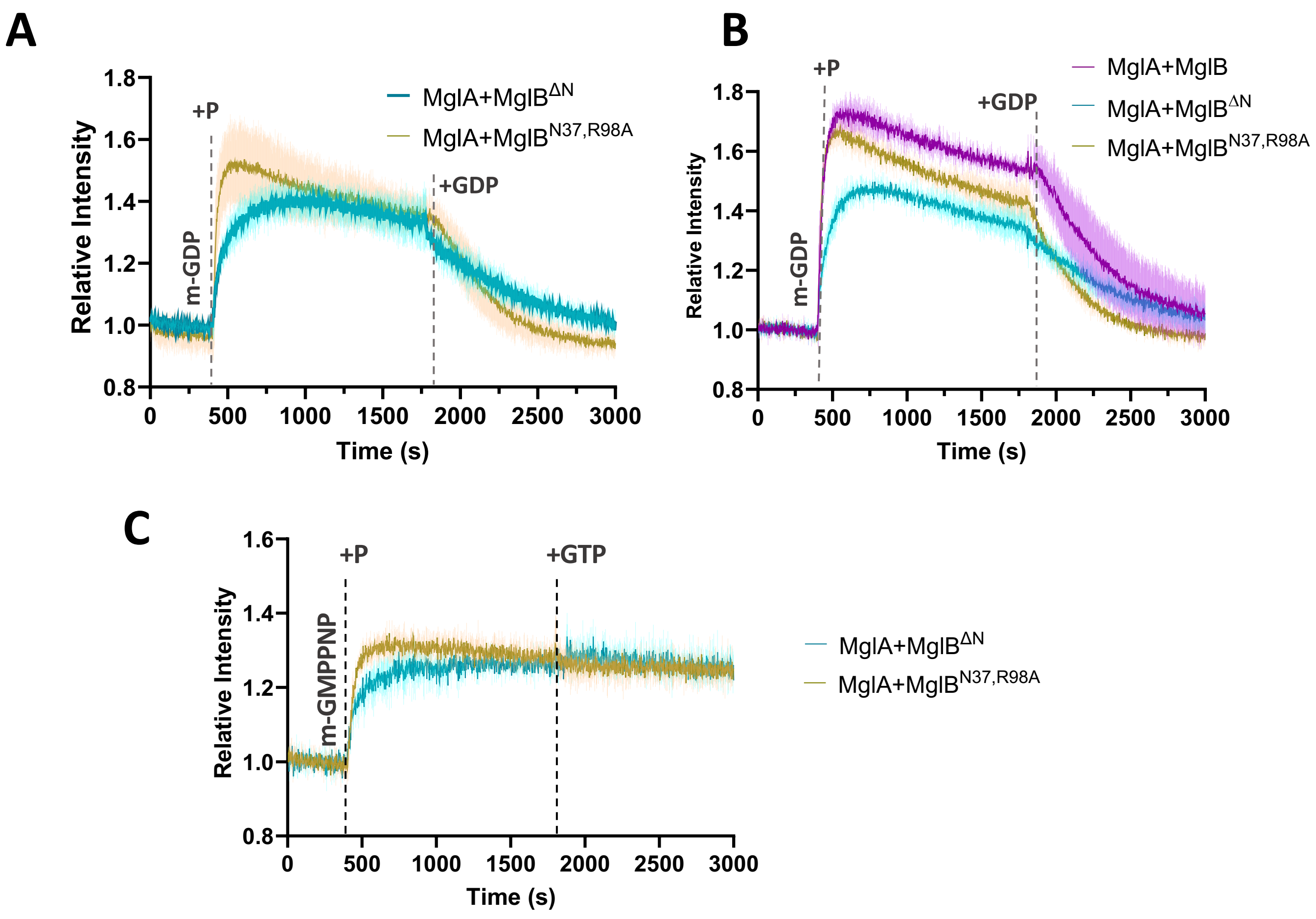

Supplementary Figure S3. Mean and standard deviation of the nucleotide exchange kinetics shown by the MglB mutants with MglA

A: Kinetics of increase in *mant*-GDP fluorescence (region labeled as *m*-GDP) upon adding MglA with MglB$^{\Delta N}$ (cyan) and MglB^{N37,R98A} (ochre) at 400 seconds (marked by dashed line labeled '+P'), followed by competition of *mant*-GDP by adding excess unlabeled GDP at 1800 seconds (marked by dashed line labeled '+GDP'). The traces represent the mean values across replicates with the respective standard deviations in shaded lines.

B: Similar plot as **A** with MglA and MglB in 1:4 ratio (purple), and the same ratio of MglB$^{\Delta N}$ (cyan) and MglB^{N37,R98A} (ochre).

C: Kinetics of increase in *mant*-GMPPNP fluorescence (region labeled as *m*-GDP) upon adding 1:2 ratio of MglA with MglB$^{\Delta N}$ (cyan) and MglB^{N37,R98A} (ochre) at 400 seconds (marked by dashed line labeled '+P'), followed by competition of *mant*-GMPPNP by adding excess unlabeled GTP at 1800 seconds (marked by dashed line labeled '+GTP'). The traces represent the mean values across replicates with the respective standard deviations in shaded lines.

Supplementary Table S1: List of primers used

**Plasmid Constructs**	**Primer Sequence**
pHis17 *mglA-link-mglB H6*	MglA-link-B-F<br>5'CCTCACCGAGCTCAAGAAGGGTGGTGGTTCCGGCACGCAACTGGTGATGTACG3'<br>MglB H6-R 5'GCTTTTAATGATGATGATGATGATGGG3'
pHis17 *mglA-link-mglB$^{\Delta Ct}$ H6*	MglA-link-B-F<br>5'CCTCACCGAGCTCAAGAAGGGTGGTGGTTCCGGCACGCAACTGGTGATGTACG3'<br>MglBΔCt H6-R<br>5'GCTTTTAATGATGATGATGATGATGGGATCCAGGACTGTCAGTCTTCT3'
pHis17 *mglB Strep*	MglB-F 5'GTTTAACTTTAAGAAGGAGATATACAT3'<br>MglB-Strep-R<br>5'TTATTTTTCGAACTGAGGATGAGACCAGGATCCCTCGCTGAAGAGGTTGTCG3'
pHis17 *mglB$^{\Delta Ct}$ Strep*	MglB-F 5'GTTTAACTTTAAGAAGGAGATATACAT3'<br>MglB-Strep-R<br>5'TTATTTTTCGAACTGAGGATGAGACCAGGATCCAGGACTGTCAGTCTTCTTC3'
pHis17<br>*mglB-Strep-Operon-mglA*	T7-F<br>5'TAATACGACTCACTATAGGG3'<br>Strep-Operon-MglA-R<br>5'GGATGAGTAATTGATGAAGGACATTGGCTTCCCGGGTTATTTTTCGAACTGAGGATGAG3'<br>Strep Operon MglA-F<br>5'FCTCATCCTCAGTTCGAAAAATAACCCGGGAAGCCAATGTCCTTCATCAATTACTCATCC3'<br>T7-R<br>5'GCTAGTTATTGCTCAGCGG3'
pHis17 *mglB$^{\Delta N}$*	MglB ΔN-F<br>5'GTTTAACTTTAAGAAGGAGATATACATATGTACGAAGAGGAGTTCACC3'<br>MglB H6-R 5'GCTTTTAATGATGATGATGATGATGGG3'
pHis17 *mglB $^{N37A\ R98A}$*	MglB N37A-F 5'CCTCGTCGACAAGGCCGGGCAGCTCATCTCC3'<br>MglB R98A-R 5'CGACCAGCACGACCGCGCTGCCGACGATGG3'
pHis17 *mglA. mglB*, *mglB$^{\Delta Ct}$*	Refer Baranwal et al., 2019
pHis17 Kan^{r}	pHisK-F<br>5'GCTAAAGTTGTTAAAGACTTTAAATTGCCGCGCGGCAGCCACATG3'<br><br>pHisK-R<br>5'GTATATATGAGTAAACTTGGTCTGACAGTTAGAAAAACTCATCGAGCATC3'
